# Effects of local reduction of endogenous α-synuclein using antisense oligonucleotides on the fibril-induced propagation of pathology through the neural network in wild-type mice

**DOI:** 10.1186/s40478-024-01766-3

**Published:** 2024-05-14

**Authors:** Tatsuhiko Sano, Tetsuya Nagata, Satoe Ebihara, Kie Yoshida-Tanaka, Ayako Nakamura, Asuka Sasaki, Aki Shimozawa, Hideki Mochizuki, Toshiki Uchihara, Masato Hasegawa, Takanori Yokota

**Affiliations:** 1 Department of Neurology and Neurological Science, Graduate School of Medical and Dental Sciences, Tokyo Medical and Dental University, 1-5-45 Yushima, Bunkyo-Ku, Tokyo, 113-8519 Japan; 2 Center for Brain Integration Research, Tokyo Medical and Dental University, 1-5-45 Yushima, Bunkyo-Ku, Tokyo, 113-8519 Japan; 3 NucleoTIDE and PepTIDE Drug Discovery Center, Tokyo Medical and Dental University, 1-5-45 Yushima, Bunkyo-Ku, Tokyo, 113-8519 Japan; 4 Department of Neurology, Osaka University Graduate School of Medicine, 2-2 Yamadaoka, Suita, 565-0871 Japan; 5 Department of Brain and Neurosciences, Tokyo Metropolitan Institute of Medical Science, 2-1-6 Kamikitazawa, Setagaya-Ku, Tokyo, 156-0057 Japan

**Keywords:** α-synuclein, Propagation, Antisense oligonucleotide, Prion, Parkinson’s disease, Synucleinopathy

## Abstract

In Parkinson's disease and other synucleinopathies, fibrillar forms of α-synuclein (aSyn) are hypothesized to structurally convert and pathologize endogenous aSyn, which then propagates through the neural connections, forming Lewy pathologies and ultimately causing neurodegeneration. Inoculation of mouse-derived aSyn preformed fibrils (PFFs) into the unilateral striatum of wild-type mice causes widespread aSyn pathologies in the brain through the neural network. Here, we used the local injection of antisense oligonucleotides (ASOs) against *Snca* mRNA to confine the area of endogenous aSyn protein reduction and not to affect the PFFs properties in this model. We then varied the timing and location of ASOs injection to examine their impact on the initiation and propagation of aSyn pathologies in the whole brain and the therapeutic effect using abnormally-phosphorylated aSyn (pSyn) as an indicator. By injecting ASOs before or 0–14 days after the PFFs were inoculated into the same site in the left striatum, the reduction in endogenous aSyn in the striatum leads to the prevention and inhibition of the regional spread of pSyn pathologies to the whole brain including the contralateral right hemisphere. ASO post-injection inhibited extension from neuritic pathologies to somatic ones. Moreover, injection of ASOs into the right striatum prevented the remote regional spread of pSyn pathologies from the left striatum where PFFs were inoculated and no ASO treatment was conducted. This indicated that the reduction in endogenous aSyn protein levels at the propagation destination site can attenuate pSyn pathologies, even if those at the propagation initiation site are not inhibited, which is consistent with the original concept of prion-like propagation that endogenous aSyn is indispensable for this regional spread. Our results demonstrate the importance of recruiting endogenous aSyn in this neural network propagation model and indicate a possible potential for ASO treatment in synucleinopathies.

**Supplementary Information:**

The online version contains supplementary material available at 10.1186/s40478-024-01766-3.

## Introduction

Α-synuclein (aSyn) is a major component of somatic and neuritic inclusions called Lewy bodies (LBs) and Lewy neurites (LNs), respectively [7, 76, 87]. LBs and LNs are found together with the neuronal loss [88] and the hallmarks in the autopsy brains of patients with sporadic [76, 87] and some familial [69] Parkinson's disease and dementia with Lewy bodies (DLB) [7]. Lewy pathologies are also found in the peripheral autonomic nervous system in association with pure autonomic failure [3]. In addition, aSyn forms glial and neuronal inclusions in the brain associated with multiple system atrophy (MSA) [5, 75, 79]. Therefore, these diseases are called synucleinopathies. The abnormal aSyn is phosphorylated [23], partially ubiquitinated [27, 48], associated with p62 [43], and deposited as fibrillar or filamentous forms [6] with cross-β structures [71] in the LBs [4].

aSyn is a 140-amino acid residue protein that is predominantly and ubiquitously abundant in the brain, in particular throughout the neocortex, hippocampus, olfactory bulb, striatum, thalamus, and cerebellum [34]. It contains three major domains: an amphipathic helix-containing N-terminal membrane-binding domain, a hydrophobic non-amyloid-beta component domain involved in aSyn aggregation, and a hydrophilic C-terminal domain with chaperone-like activity. Although its physiological function has not been fully elucidated, aSyn is known to be abundant in the presynapse and interact with synaptic vesicles to modulate vesicle recycling physiologically. Point mutations [39, 42, 62, 63, 90] and multiplication [18, 32, 73] of the *SNCA* gene, which encodes aSyn, have been identified as causative genetic abnormalities in familial PD and DLB. Thus, structural changes or overexpression of aSyn protein are relevant to the development of familial PD and DLB.

In brains with sporadic PD, aSyn pathology emerges from the olfactory bulb and/or brainstem and spreads to other brain regions concurrently with the progression of disease symptoms [15]. Similar neuropathological spreads have been reported in other neurodegenerative diseases, such as Alzheimer's disease [13] and amyotrophic lateral sclerosis [65]. Studies conducted in vitro [51, 57, 84], in experimental animals [49, 50, 54, 55, 64], and in patients [41, 46, 47, 78] have suggested that the cause of the pathological progression may be prion-like aSyn transmission, in which pathogenic misfolded aSyn triggers normal endogenous aSyn aggregation. This prion-like propagation of aSyn pathology is thought to be mediated by the uptake of misfolded aSyn by micropinocytosis [30] or receptor-mediated endocytosis [53] into the cell, where it recruits endogenous aSyn [37, 85] and induces its aggregation by converting normal endogenous monomers into abnormally-phosphorylated aggregates (intracellular propagation). Pathogenic aSyn aggregates may be released by exocytosis from the affected neuron [24, 35], reabsorbed by the synaptic terminals of other neurons, and spread via the neural networks primarily in a synaptic retrograde manner (network propagation), as previously reported [29, 33, 38, 49, 54, 59, 72, 83]. It suggests that treatment to reduce endogenous aSyn production may be beneficial in patients with PD and other synucleinopathies.

Antisense oligonucleotides (ASOs) can specifically induce the degradation of target mRNA or modulation of splicing, resulting in a decrease or increase in the corresponding protein [8, 77]. In recent years, ASO drugs have been launched for spinal muscular atrophy [19], hereditary transthyretin amyloidosis [9], and Duchenne muscular dystrophy [31, 56, 66], which are all progressive degenerative diseases. To date, several antibodies [68, 70, 89] and a vaccine [45] have been developed in clinical trials, targeting different forms and structural states of aSyn. Although these approaches can eliminate pathological aSyn aggregates transmitted from cell to cell, they may be ineffective when targeting intracellular LBs and LNs. Treatment with ASOs can target intracellular mRNA and may overcome the limitations of antibody therapies by reducing endogenous aSyn protein levels. ASO therapies are already being investigated for PD and DLB treatment in animal models including the propagation model [10, 20, 82] and clinical trials are currently being conducted by IONIS therapeutics (NCT03976349 and NCT04165486). As for the propagation model, the initiation [20] and progression of abnormal aSyn pathology have been reported to be inhibited by *Snca* mRNA-targeting ASOs in WT mouse models of prion-like aSyn pathological propagation [10, 20] induced by inoculation of recombinant aSyn preformed fibrils (PFFs).

To evaluate the ASO effect against intracellular and network propagation of aSyn pathologies, here we chose to inject ASOs intrastriatally rather than intracerebroventricularly (ICV) in order to control the area of gene knockdown and reduction in aSyn. We used a WT mouse, aSyn PFFs intrastriatal inoculation model to examine the effects of *Snca* ASO injection timing and locations on the initiation and propagation of abnormal aSyn pathology. *Snca* ASOs showed different effects on aSyn prion-like pathological propagation depending on the conditions of injection.

## Materials and methods

### Mice

7 weeks WT C57BL/6 J female mice (Oriental Yeast) were maintained on a 12 h light/dark cycle in a pathogen-free animal facility with free access to food and water. All experimental protocols were approved by the Institutional Animal Care and Use Committee of Tokyo Medical and Dental University (Approval Number A2022-085A).

### Oligonucleotide synthesis

All the chemically-modified oligonucleotides used in the experiment were purchased from GeneDesign and are described in Additional file 1: Table S1.

### Antibodies

The antibodies used in this study are presented in Additional file 1: Table S2. The anti-phosphorothioate (PS) antibody [39] was kindly provided by Ionis Pharmaceuticals.

### Preparation of recombinant aSyn fibrils

Recombinant mouse WT aSyn fibrils were prepared as described previously [54]. Briefly, purified aSyn was incubated at 37 °C in a shaking incubator at 200 rpm in 30 mM Tris–HCl (pH 7.5) containing 0.1% NaN3 for 72 h. aSyn fibrils were pelleted by spinning the assembly mixtures at 113,000×*g* for 20 min, resuspended in 30 mM Tris–HCl buffer (pH 7.5), and sonicated for 3 min. The protein concentrations were determined by high-performance liquid chromatography (HPLC). aSyn fibrils were re-sonicated for 60 s prior to injections.

### Stereotaxic surgery

Seven to 9-week-old female mice positioned on the stereotaxic instrument (SR-5 M, Narishige) under anesthesia with isoflurane (Wako) were unilaterally injected with 10 μg of sonicated recombinant mouse aSyn fibrils into the left striatum (Lt Str A-P: 0.5 mm; M-L: − 2.0 mm; D-V:  − 3.0 mm), with 300 μg antisense oligonucleotides into the left or right striatum (Rt Str A-P: 0.5 mm; M-L: 2.0 mm; D-V:  − 3.0 mm), or with phosphate-buffered saline (PBS) into the unilateral striatum using a microsyringe (1702RN, Hamilton) in the timeline described. All mice were sacrificed under anesthesia with isoflurane. After perfusion with PBS, the brains were collected. For biochemical analysis, brains were snap-frozen with liquid nitrogen and stored at − 80 °C. For histopathological analysis (silver staining, immunohistochemistry with anti-pSyn (pSer129), anti-ubiquitin, and anti-p62 antibodies, and immunofluorescence staining), brains were fixed in a 10% neutral-buffered formalin solution (Wako) and then cut on a vibratome (VT1000S, Leica) at a 50 μm thickness. For RNAscope in situ hybridization and immunohistochemistry with the anti-PS antibody, after subsequent perfusion with 4% paraformaldehyde, fixed brains were paraffin-embedded and sectioned (5 μm thickness) and placed on SuperFrost Plus slides (Thermo Fischer Scientific).

### Quantitative real-time polymerase chain reaction (PCR) assay

RNA was extracted from mouse brains by using the MagNA Pure 96 system (Roche Diagnostics) and reverse-transcribed (RT) with PrimeScript RT Master Mix (Takara Bio). To measure the relative RNA expression, qRT-PCR analysis was conducted using the Light Cycler 480 Real-Time PCR Instrument (Roche Diagnostics). The primers and probes for qRT-PCR are shown in Additional file 1: Table S3. After being measured and normalized to *Actb* mRNA levels, the RNA expression levels were presented as a percentage of the PBS vehicle treatment group and expressed as mean ± SEM. All qRT-PCR studies were conducted in accordance with the Minimum Information for Publication of Quantitative Real-time PCR Experiments (MIQE) guidelines [16].

### Preparation of total lysates and sarkosyl-insoluble fractions of mouse brains and immunoblotting

For the total lysates, frozen mouse brains were homogenized on ice in lysis buffer (50 mM N-2-hydroxyethylpiperazine-N’-2-ethanesulfonic acid (HEPES, pH 7.4), 150 mM NaCl, 5 mM MgCl2, 10% glycerol, 1% Triton X-100, and cOmplete EDTA-free protease inhibitor cocktails (Roche Diagnostics)). The lysates were centrifuged at 10,000×*g* for 10 min at 4 °C. The supernatants were collected and then sonicated for 20 s. After determining the protein concentration using a bicinchoninic acid Protein Assay Kit (Thermo Fisher Scientific), ethylene glycol tetraacetic acid (EGTA) was added to the final concentration of 1 mM. Samples were adjusted to the final concentration of 0.5 μg/μL. For sarkosyl-soluble and -insoluble fractions, frozen mouse brains were homogenized in 20 volumes (w/v) of A68 buffer (10 mM Tris–HCl (pH 7.5) containing 10% sucrose, 0.8 M NaCl, 1 mM EGTA, cOmplete EDTA-free protease inhibitor cocktails (Roche Diagnostics), and PhosSTOP phosphatase inhibitor (Roche Diagnostics)) and then incubated for 30 min at 37 °C after addition of sarkosyl (final concentration, 2%). The homogenates were centrifuged at 9,500×*g* for 10 min at 25 °C, and then the supernatants were collected and ultracentrifuged at 126,000×*g* for 20 min at 25 °C. The supernatants were collected as the sarkosyl-soluble fraction. The pellets were washed with saline and ultracentrifuged as before. The resulting pellets were collected as the sarkosyl-insoluble fraction of the mouse brains, resuspended in 3 volumes (w/v) 30 mM Tris–HCl (pH 7.5), and sonicated for 30 s. The lysates were added to the Laemmli sample buffer and heated at 100 °C for 5 min. Then, 5 to 10 μL samples were loaded on 15% polyacrylamide gels (ATTO), electrophoresed, transferred to PVDF membranes (Bio-Rad), and detected using the anti-pSyn (pSer129 #64) antibody. After the final wash, immunoreactive bands were visualized with SuperSignal Westdura Extended Duration Substrate (Thermo Fisher Scientific) and detected by Chemidoc (Bio-Rad). The band intensity of the immunoblots was quantified using Fiji (NIH) by the background subtraction method described previously [25].

### In situ hybridization (ISH) by RNAscope

In situ hybridization was performed using the RNAscope 2.5 HD Brown Chromogenic Reagent Kit according to the manufacturer’s instructions (Advanced Cell Diagnostics [ACD]). Slides were baked in the oven for 1 h at 60 °C, and then incubated twice in xylene for 5 min and washed in 100% ethanol twice for 1 min. Sections were incubated in hydrogen peroxide for 10 min at room temperature, then washed with distilled water, and boiled with 1 × RNAscope target retrieval buffer for 15 min. Slides were then washed in distilled water, transferred to 100% ethanol for 3 min, and then allowed to dry. Protease Plus treatment was applied to the tissues, incubated in the oven at 40 °C for 30 min, and washed in distilled water twice for 2 min. Slides were incubated with *Snca* probes (ACD#313,281) for 2 h at 40 °C. Further amplification of the target probe signal was performed according to the manufacturer’s instructions (RNAscope 2.5 HD detection protocol Amp 1–6). The hybridization signals were detected with 3,3’-diaminobenzidine (DAB) staining. For quantification of *Snca* mRNA expression, images were acquired at 20 × magnifications using the slide scanner (VS200, Olympus). DAB signals within the same (294 μm × 294 μm) area square in each brain area were binarized automatically using Fiji (NIH). The density of the signal positive area was quantified.

### Immunohistochemistry of anti-PS antibody

The slides were deparaffinized in xylene, pretreated for antigen retrieval using proteinase K (Dako), and incubated at room temperature (25 °C) for 5 min. The samples were incubated in BLOX ALL (Vector) to block endogenous peroxidase activity. The slides were blocked using Background Buster (Innovex Biosciences) for 30 min. A polyclonal rabbit anti-PS antibody [40] was then applied to the slides at a dilution of 1:15,000 (diluted in 10% bovine serum albumin), and the slides were incubated at room temperature (25 °C) for 1 h. After three washes in PBS, the slides were incubated with donkey anti-rabbit horseradish peroxidase-conjugated secondary antibody (Jackson ImmunoResearch Laboratories) at a 1:200 dilution for 30 min and subsequently developed with DAB and counterstained with hematoxylin. We used these solutions as the primary antibody for anti-PS immunohistochemical staining.

### Silver staining

Brain sections were stained using Campbell-Switzer [17, 80] and Gallyas-Braak [14, 26] silver as previously described.

### Immunohistochemistry of anti-pSyn (pSer129), anti-ubiquitin, anti-p62, anti-Iba1, anti-NeuN, and anti-GFAP antibodies and quantification of pSyn (pSer129)-positive cells

For anti-pSyn (pSer129), anti-ubiquitin, and anti-p62 immunostaining, Brain sections were treated with 98% formic acid, washed, and boiled in 20 mM Tris–HCl buffer (pH 9.0) at 100 °C for 20 min. For anti-NeuN immunostaining, the sections were autoclaved for 20 min at 121℃ for 20 min. The sections were then incubated with 3.0% H_2_O_2_ in methanol to inactivate endogenous peroxidases, treated with 0.5% Triton X-100 in PBS, blocked with 10% calf serum in PBS, and immunostained with appropriate antibodies. After incubation with the biotinylated-secondary antibody (Vector), biotin labeling was detected using the ABC staining kit (Vector) and counterstained with hematoxylin. For quantification of cells, brain sections approximately 0.5 mm anterior to the bregma for evaluation of the striatum (caudate putamen), primary motor cortex and corpus callosum; approximately 1.7 mm posterior to the bregma for evaluation of the primary somatosensory cortex (primary somatosensory barrel cortex), amygdala (basomedial amygdala), and thalamus (paraventricular nucleus); and approximately 3.1 mm posterior to the bregma for evaluation of the substantia nigra (substantia nigra pars compacta) were immunostained for pSer129. Each brain area was identified by reference to the Mouse Atlas (Allen Brain Atlas, Allen Institute; http://atlas.brain-map.org/). The slide of each brain area was observed at 20 × magnifications with a slide scanner (VS200, Olympus) using Virtual-Z scan mode to acquire z-stacks within images (z-width 1.18 μm, 43 continuous cross-sections images). For quantification of pSer129-positive cells, inclusion localization was defined by hematoxylin staining nuclear and cell body outlines. Neuritic inclusions were positive for thread-like structures that are contiguous in two or more cross-sections. The number of cells with pSyn (pSer129)-positive somatic or neuritic inclusions within the same (294 μm × 294 μm) area square was counted to quantify cells specifically within the brain area of interest. Because it is difficult to accurately observe neuritic inclusions in the presence of somatic inclusions, cells with both somatic and neuritic inclusions or with only somatic inclusions were counted as somatic pSyn-positive cells. Cells with only neuritic inclusions were counted as neuritic pSyn-positive cells.

### Immunofluorescent staining

For double-label immunofluorescence, brain sections were washed, boiled at 100 °C for 20 min, treated with 0.5% Triton-X 100 in PBS, blocked with 10% calf serum in PBS, and incubated overnight at 4 °C in a cocktail of appropriate antibodies. The sections were then washed and incubated in a cocktail of Alexa Fluor 594-conjugated donkey anti-rabbit IgG (Invitrogen) and Alexa Fluor 488-conjugated donkey anti-mouse IgG (Invitrogen). After further washing, sections were coverslipped with VECTASHIELD Mounting Medium with DAPI (Vector) and observed with a laser-scanning confocal fluorescence microscope (A1R, Nikon).

### Behavioral analysis

Behavioral tests were conducted between 8:00 a.m. and 12:00 a.m. in a bright room. All devices were cleaned with 70% ethanol before use to exclude odor effects.

### Wire hang tests

Wire hang tests were conducted the day before the sacrifice. The mouse was placed on a wire mesh, and the examiner gently shook it so that the mouse grabbed the mesh and then flipped it over. The time to fall was measured using 900 s as a threshold. Measurements were taken twice in 15 min intervals, and the average value was used in the analysis.

### Rotarod tests

Acclimation for the rotarod test was conducted from three days before the sacrifice until the day of the sacrifice. Every other day during the acclimation period, the mice were placed on the rotarod apparatus and trained in acceleration mode, accelerating from 4 to 40 rpm in 5 min, four times each at 5 min intervals [21]. The test was conducted one hour after the last acclimation on the day of the sacrifice. The test was performed twice in a row in the same.

### Statistical analysis

Prism 9 (GraphPad Software) was used to analyze the data for cell counting of immunohistochemistry, behavioral analysis, qRT-PCR and immunoblotting. Data are presented as mean ± SEM. Differences between the two groups were analyzed by unpaired two-tailed Student’s t-test. Differences among more than three groups were analyzed by one-way ANOVA with Tukey’s test.

## Results

### Suppression of endogenous *Snca* gene expression by intrastriatal injection of *Snca* ASOs in WT mouse brain

**Fig. 1:**
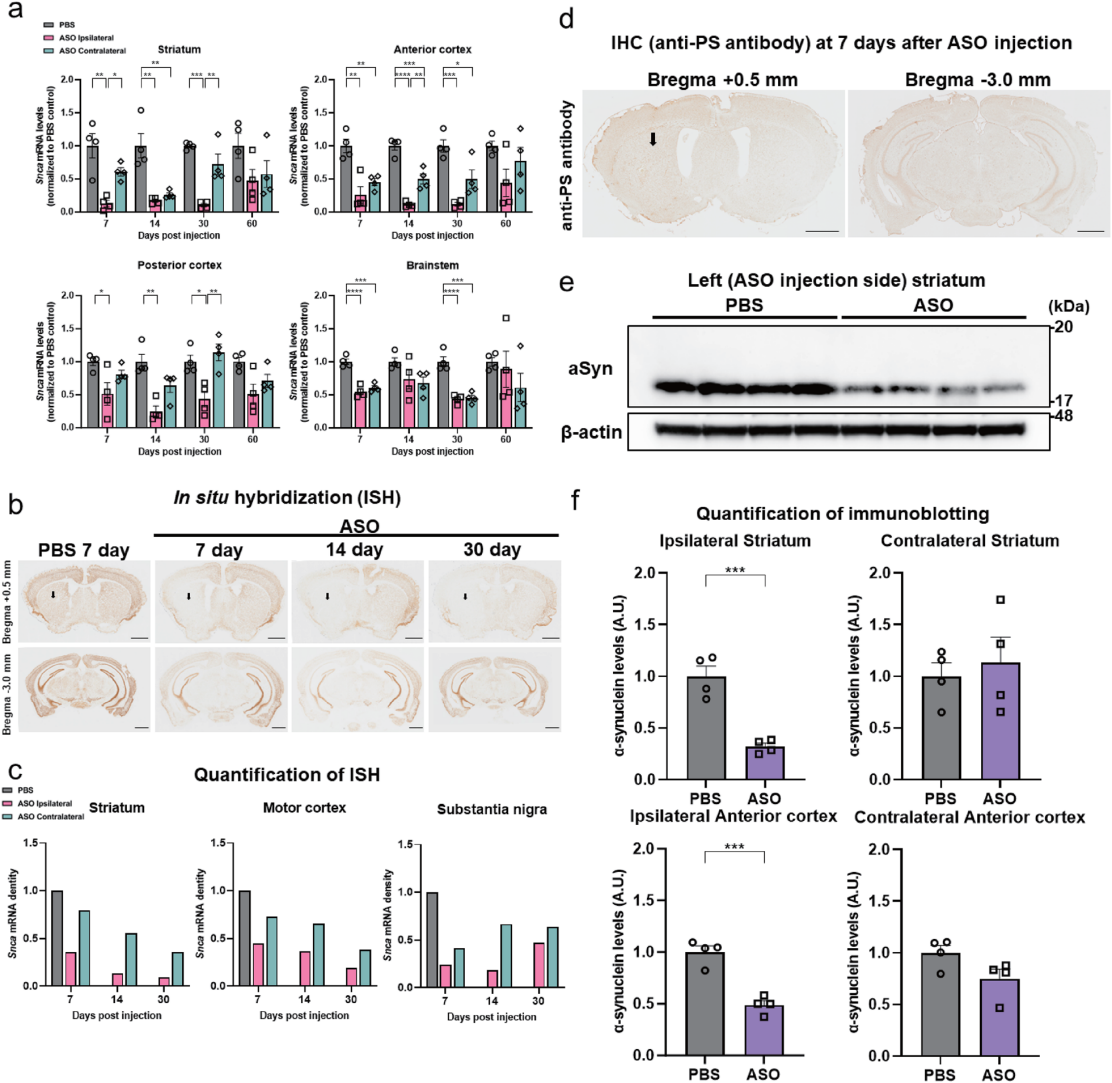
Intrastriatal injection of antisense oligonucleotides (ASOs) against the *Snca* gene efficiently downregulated the levels of endogenous *Snca* mRNA and α-synuclein (aSyn) protein in the brain, predominantly on the injection side. **a** Time course of *Snca* mRNA expression in the striatum, anterior cortex, posterior cortex, and brainstem on each side of the brain after intrastriatal injection of phosphate-buffered saline (PBS) or 300 μg of *Snca* ASOs by qRT-PCR (n = 4 per each group; *P < 0.05, **P < 0.01, ***P < 0.001, ****P < 0.0001) at 7, 14, 30, or 60 days. ASOs efficiently downregulated the levels of endogenous *Snca* mRNA predominantly on the injection side. **b** Time course of *Snca* mRNA expression at 0.5 mm anterior to the bregma and 3.0 mm posterior to the bregma by i*n situ* hybridization at 7 days after left intrastriatal injection of PBS and 7, 14, or 30 days after left intrastriatal injection of 300 μg of *Snca* ASOs. The arrows indicate the injection site of PBS or *Snca* ASOs. The scale bar represents 1 mm. **c** Quantification of the *Snca* mRNA expression revealed by in situ hybridization (shown in Fig. 1b) at the striatum, anterior cortex, and substantia nigra on each side of the brain (n = 1). The density of the probe signals was normalized by that measured in the PBS-injected animal.** d** Distribution of *Snca* ASOs by anti-phosphorothioate (PS) antibody immunostaining at 0.5 mm anterior to and 3.0 mm posterior to the bregma at 7 days after intrastriatal injection of 300 μg of *Snca* ASOs. The arrow indicates the injection site of *Snca* ASOs. The scale bar represents 1 mm. **e** Immunoblotting of aSyn protein from the ipsilateral striatum at 14 days after intrastriatal injection of 300 μg of *Snca* ASOs. **f** Quantification of aSyn protein from the striatum and anterior cortex on each side of the brain at 14 days after intrastriatal injection of 300 μg of *Snca* ASOs by immunoblotting (n = 4 per each group; ***P < 0.001). The aSyn levels are shown as a ratio to the average of that measured in the PBS-injected group. ASOs efficiently downregulated the levels of endogenous aSyn protein predominantly on the injection side. “Ipsilateral (contralateral)” corresponds to a region of the brain ipsilateral (contralateral) to the ASO injection side. The “anterior (posterior) cortex” corresponds to the anterior (posterior) half of the cortex. The “motor cortex” corresponds to the primary motor cortex. All data are expressed as mean ± SEM

To understand the site-specific spread of the gene knockdown effect by the local injection, we first performed intrastriatal injections of *Snca* ASOs to achieve suppression of the endogenous *Snca* gene expression localized near the site of injection in the brain of the 7 week-old WT female mouse (n = 4). *Snca* ASOs showed a dose-dependent suppressive effect on *Snca* mRNA in the striatum (Additional file 1: Fig. 1a) without suppressing *Sncb* and *Sncg* mRNA (Additional file 1: Fig. 2), while scrambled ASOs showed no suppressive effect (Additional file 1: Fig. 1b). For sufficient suppression of the endogenous *Snca* gene expression, 300 µg of ASOs were injected in the following experiments. The *Snca* mRNA suppression effect reached approximately 87% in the left striatum, the site of *Snca* ASO injection, compared to approximately 39% in the right striatum 7 days after the injection of *Snca* ASOs (Fig. 1a), a side difference that was consistent with previous reports [74]. Suppression effects of *Snca* mRNA were also observed in the anterior (approximately 74% on the left and 55% on the right) and the posterior (approximately 49% on the left and 19% on the right) half of the cortex with differential tendencies by side, but there was no difference between sides in the brainstem (approximately 45% on the left and 39% on the right) (Fig. 1a). The suppressive effect persisted for more than 30 days (Fig. 1a). ISH to *Snca* mRNA was also performed in 7-week-old WT female mice (n = 1) and the results were consistent with those of qRT-PCR (Fig. 1b, c). Immunostaining with anti-PS antibody using the serial section of brain prepared for ISH showed a predominant distribution of *Snca* ASOs on the injection side (Fig. 1d) in 7-week-old WT female mice (n = 1), consistent with the observed suppression effect of *Snca* mRNA (Fig. 1b, c). The results we obtained from ISH and anti-PS antibody immunostaining revealed that ASOs spread around the site of the injection site with a greater uptake [74, 91] and appeared to mainly distribute by passive diffusion. Consistent with these results, the decrease in aSyn protein expression was localized around the injection site, as previously reported [74, 91]. To confirm the reduction of aSyn protein levels, immunoblotting was conducted on the brain 14 days after *Snca* ASO injection. Approximately 68% of aSyn protein reduction was observed in the left striatum and approximately 51% in the left anterior half of the cortex, whereas no reduction in aSyn protein was observed in the brain counterparts on the right side (Fig. 1e, f).

### Prevention of the appearance of aSyn pathologies by ***Snca*** ASO pre-treatment at the aSyn PFFs inoculation site

**Fig. 2:**
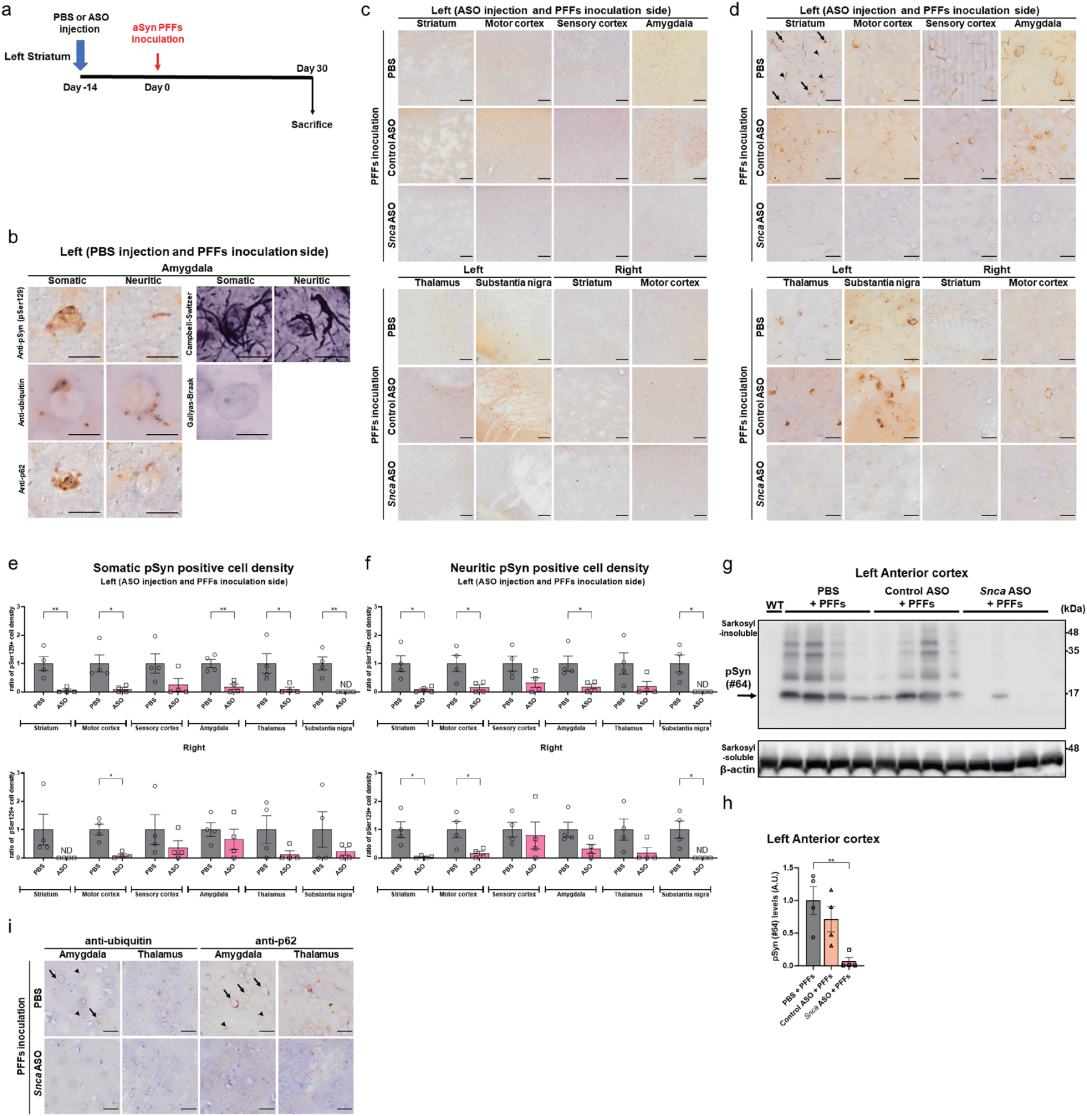
*Snca* ASO ipsilateral pre-treatment prevented the progression of abnormally-phosphorylated aSyn (pSyn) pathology in most brain areas. **a** Timeline for a single 300-μg intrastriatal ipsilateral ASO injection 14 days prior to aSyn preformed fibrils (PFFs) inoculation with termination 30 days after PFFs inoculation. **b** High-magnification images of cells with somatic and neuritic inclusions in the left amygdala of the PBS-pre-treated and PFFs-inoculated animals by immunostaining with anti-pSyn (pSer129), anti-ubiquitin, and anti-p62 antibodies and Campbell-Switzer and Gallyas-Braak silver staining. The scale bar represents 10 μm. **c** Low magnification images of pSyn (pSer129)-positive inclusions in the left striatum, motor cortex, sensory cortex, amygdala, thalamus, substantia nigra, and the right striatum and motor cortex 30 days after PFFs inoculation and PBS, Control ASO or *Snca* ASO injection 14 days before. The scale bar represents 100 μm. **d** Representative images of pSyn (pSer129)-positive somatic (arrows) and neuritic (arrowheads) inclusions in the left striatum, motor cortex, sensory cortex, amygdala, thalamus, substantia nigra, and the right striatum and motor cortex 30 days after PFFs inoculation and PBS, Control ASO or *Snca* ASO injection 14 days before. The scale bar represents 20 μm. **e, f** The ratio of cells with pSyn (pSer129)-positive somatic (**e**) and neuritic (f) inclusions density in the striatum, motor cortex, sensory cortex, amygdala, thalamus, and substantia nigra on each side of the brain (n = 4 per each group; *P < 0.05, **P < 0.01). In the *Snca* ASO pre-treatment group compared to the PBS pre-treatment groups, there were a reductions in the density of pSyn-positive cells with both types of somatic and neuritic inclusions in the left striatum and other brain areas. The ratio of pSyn (pSer129)-positive cell density to the average of that measured in the PBS-treated group is shown. **g** Immunoblotting of pSyn (pSer129 #64) protein in the sarkosyl-insoluble fraction extracted from the left anterior cortex at 30 days after intrastriatal injection of PFFs. The arrow indicates pSyn monomer bands. **h** Quantification of pSyn (pSer129 #64) protein in the sarkosyl-insoluble fraction from the left anterior cortex at 30 days after intrastriatal injection of aSyn PFFs (n = 4 per each group, **P < 0.01). Significant reductions in pSyn protein levels were observed in the *Snca* ASO group compared to the PBS pre-treatment groups. The pSyn levels are shown as the ratio of the band signal intensity to the average of that measured in the PBS-treated group. **i** Representative images of somatic (arrows) and neuritic (arrowheads) inclusions by immunostaining of anti-ubiquitin and anti-p62 antibodies in the left amygdala and thalamus. The scale bar represents 20 μm. “Anterior cortex” means the anterior half of the cortex. The “motor (sensory) cortex” corresponds to the primary motor (somatosensory) cortex. ND means not detected. All data are expressed mean ± SEM

To evaluate the effect of *Snca* ASO local injection prior to the initiation of aSyn protein aggregation, we injected *Snca* ASOs 2 weeks prior to aSyn PFFs inoculation into the left striatum of 7-week-old WT female mouse (n = 4) (Fig. 2a). Staining for pSyn (pSer129) revealed two types of neurons with different pSer129-immunoreactive signals in the non-treated (PBS) group. One type of neuron showed the inclusions stained with pSer129 in the somata, as in human LBs. The other type showed inclusions stained with pSer129 were observed in neurites, similar to those found in human LNs (Fig. 2b). Both types of inclusions were positive for ubiquitin and p62-staining, which are features of intracellular inclusion bodies. They were also positive for Campbell-Switzer silver staining and negative for Gallyas-Braak staining, which is the same staining property as human LBs and LNs [81] (Fig. 2b). Several reports have reported differences in sensitivity to PFFs inoculation depending on the neuronal subtype [29, 52].

In the following, we evaluated separately “somatic inclusion” and “neuritic inclusion.” Consistent with previous reports [49, 54], robust pSyn-positive cells were found in the left striatum and the brain areas with neural connections to the striatum in the PBS pre-treatment and Control ASO pre-treatment groups (Fig. 2c–f and Additional file 1: Fig. 3). In the *Snca* ASO pre-treatment group compared to the PBS pre-treatment groups, there was a reduction in the density of pSyn-positive cells with both somatic (by approximately 95%) and neuritic (by approximately 92%) inclusions in the left striatum and many other brain areas (Fig. 2e, f. Among those areas, the reduction effects in the right primary somatosensory cortex (of approximately 80% in somatic inclusions and 21% in neuritic inclusions) and amygdala (of approximately 34% in somatic inclusions and 68% in neuritic inclusions) seemed weaker (Fig. 2e, f. *Snca* ASO pre-treatment decreased pSyn-positive inclusions in tyrosine hydroxylase (TH)-positive neurons in the left substantia nigra as shown by double-label immunofluorescence (Additional file 1: Fig. 4). We also confirmed the significant reduction of pSyn protein levels by immunoblotting using the sarkosyl-insoluble fraction (by approximately 93%) in the *Snca* ASO group compared to the PBS pre-treatment groups (Fig. 2g, h). Ubiquitin and p62 immunostaining also showed a reduction in the density of cells with somatic and neuritic inclusions in the *Snca* ASO group (Fig. 2i). No cytotoxicity, immune response elicitation (Additional file 1: Fig. 5), or motor dysfunction (Additional file 1: Fig. 6) was evident after preadministration of *Snca* ASO, Control ASO or PBS.

### Inhibition of the progression of pSyn pathologies by *Snca* ASO simultaneous or post-treatment at the aSyn PFFs inoculation site

**Fig. 3:**
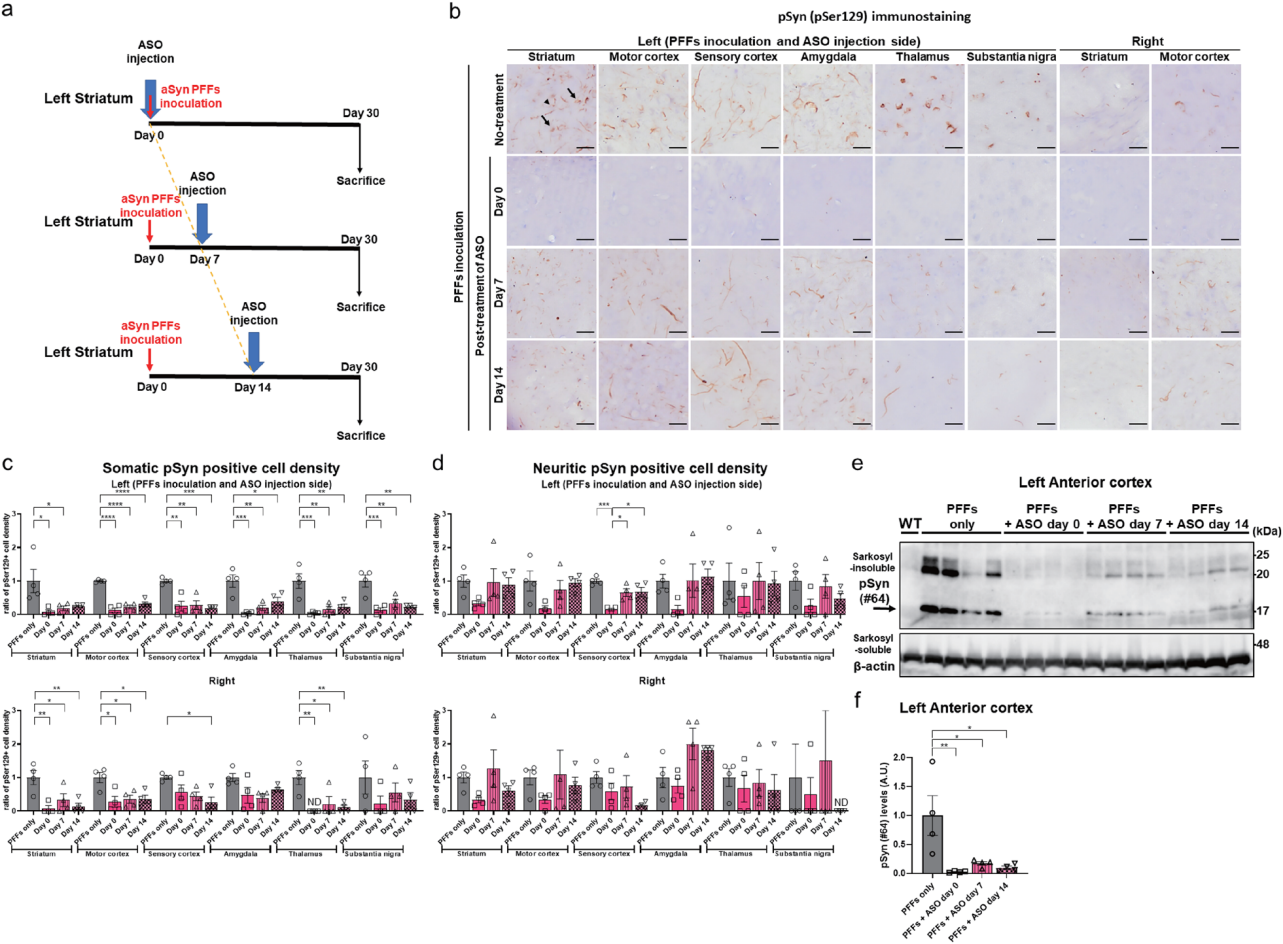
*Snca* ASO ipsilateral simultaneous- or post-treatment inhibited the progression of pSyn pathology over time up to 14 days in a time-dependent manner. **a** Timeline for a single 300 μg intrastriatal ipsilateral ASO injection after aSyn PFFs inoculation with termination 30 days after PFFs inoculation. **b** Representative images of pSyn (pSer129)-positive somatic (arrows) and neuritic (arrowheads) inclusions in the left striatum, motor cortex, sensory cortex, amygdala, thalamus, substantia nigra, and the right striatum and amygdala 30 days after PFFs inoculation and *Snca* ASO injection at the same time (day 0) as the PFFs injection or 7 or 14 days later. The scale bar represents 20 μm. **c, d** The ratio of cells with pSyn (pSer129)-positive somatic (**c**) and neuritic (**d**) inclusions density in the striatum, motor cortex, sensory cortex, amygdala, thalamus, and substantia nigra on each side of the brain (n = 4 per each group; *P < 0.05, **P < 0.01, ***P < 0.001, ****P < 0.0001). Reductions in the density of cells with both types of inclusions were observed in the left striatum and the other brain areas, including the right side by simultaneous *Snca* ASO treatment. In the *Snca* ASO post-treatment groups, there were also significant reductions of pSyn-positive somatic inclusions in the left striatum and the other brain areas. The ratio of pSyn (pSer129)-positive cell density to the average of that measured in the PFFs inoculation-only group is shown. **e** Immunoblotting of pSyn (pSer129 #64) protein in the sarkosyl-insoluble fraction extracted from the left anterior cortex 30 days after intrastriatal inoculation of PFFs. The arrow indicates pSyn monomer bands. **f** Quantification of pSyn (pSer129 #64) protein in the sarkosyl-insoluble fraction from the left anterior cortex 30 days after intrastriatal inoculation of PFFs (n = 4 per each group; *P < 0.05, **P < 0.01). pSyn levels in the sarkosyl-insoluble fraction were completely reduced in the simultaneous *Snca* ASO treatment groups and were also significantly observed in the post-treatment groups. The pSyn levels are shown as the ratio of the band signal intensity to the average of that measured in the PFFs inoculation-only group. The “anterior cortex” corresponds to the anterior half of the cortex. The “motor (sensory) cortex” corresponds to the primary motor (somatosensory) cortex. ND means not detected. All data are expressed mean ± SEM

To determine if post-treatment of *Snca* ASOs could ameliorate aSyn aggregation and pathological propagation, we next injected *Snca* ASOs simultaneously or after PFFs inoculation into the left striatum of 7-week-old WT female mouse (n = 4) (Fig. 3a). In the *Snca* ASO simultaneous treatment group compared to the PFFs-only group, there were reductions in the density of cells with pSyn-positive somatic (by approximately 96%) and neuritic inclusions (by approximately 70%) in the left striatum and the other brain areas, including those on the right side (Fig. 3b–d). In the *Snca* ASO post-treatment groups, there were also significant reductions in the density of cells with pSyn-positive somatic inclusions in the left striatum and the other brain areas (by approximately 81% and 79% in the *Snca* ASO day 7 and day 14 post-treatment groups, respectively) compared to the PFFs-only group (Fig. 3b, c). *Snca* ASO injection timing-dependent suppression of pSyn pathologies was observed in several brain areas on the left side, including the striatum, motor cortex, and amygdala. By contrast, no reduction was observed in the density of cells with pSyn-positive neuritic inclusions following *Snca* ASO post-treatment more than 7 days after PFFs inoculation (Fig. 3b, d). Consistent with the somatic pSyn pathologies, immunoblotting showed that pSyn in the sarkosyl-insoluble fraction was completely reduced (by approximately 98%) in the *Snca* ASO simultaneous treatment compared to the PFFs-only group (Fig. 3e, f). The reducing effects (of 83% and 90% in the *Snca* ASO day 7 and day 14 post-treatment groups, respectively, compared to the PFFs-only group) were also significantly observed up to 14 days after PFFs inoculation (Fig. 3e, f).

### Suppression of the appearance of pSyn pathologies by *Snca* ASO pre-treatment at the distant site of aSyn PFFs inoculation

**Fig. 4:**
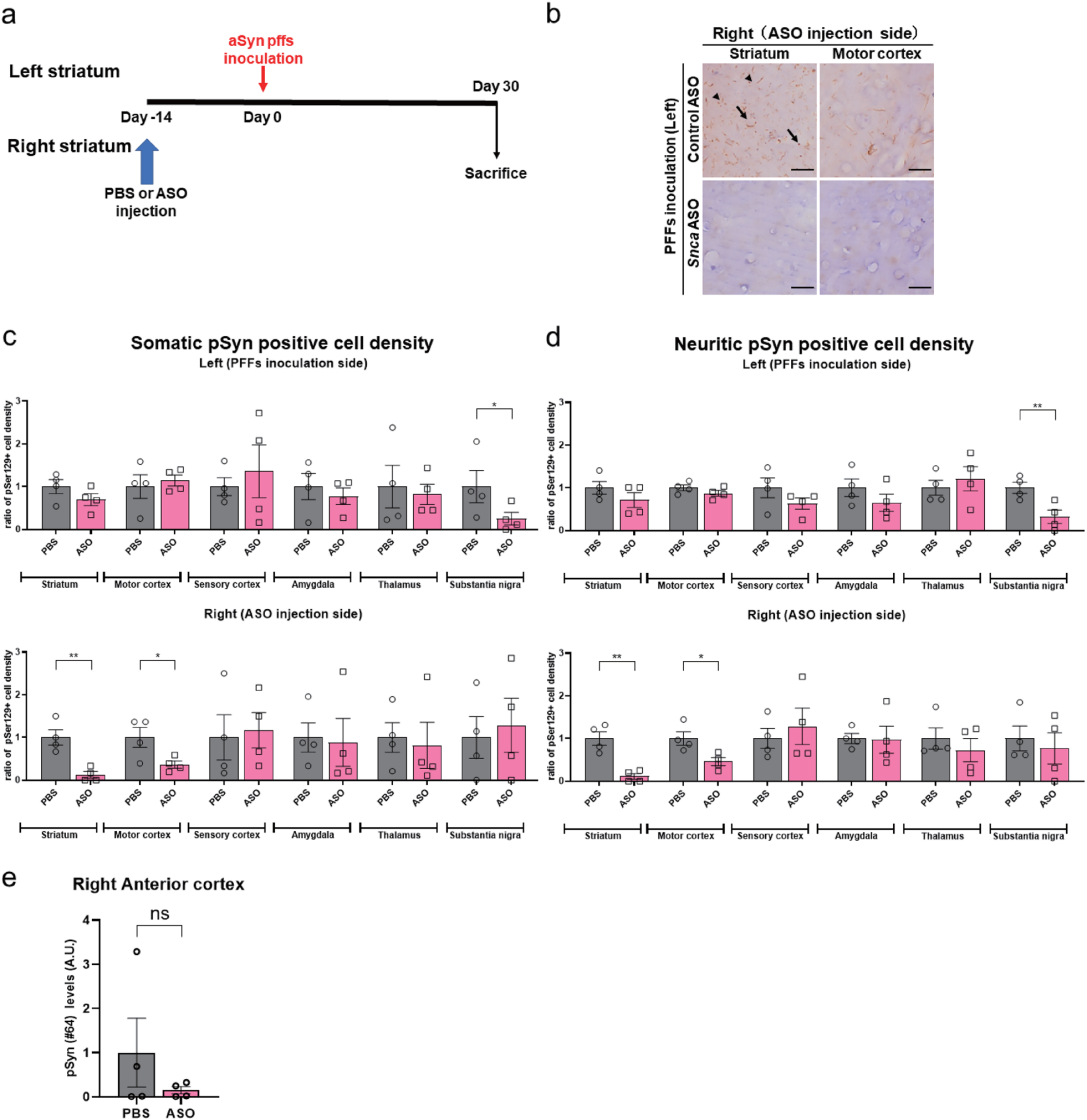
*Snca* ASO contralateral pre-treatment locally inhibited the progression of pSyn pathology in the vicinity of ASO injection. **a** Timeline for a single 300-μg intrastriatal contralateral ASO injection prior to aSyn PFFs injection with termination 30 days after PFFs inoculation. **b** Representative images of pSyn (pSer129)-positive somatic (arrows) and neuritic (arrowheads) inclusions in the right striatum and motor cortex 30 days after PFFs inoculation and PBS or *Snca* ASO treatment 14 days prior to the PFFs inoculation. The scale bar represents 20 μm. **c, d** The ratio of cells with pSyn (pSer129)-positive somatic (**c**) and neuritic (**d**) inclusions density in the striatum, motor cortex, sensory cortex, amygdala, thalamus, and substantia nigra on each side of the brain (n = 4 per each group; *P < 0.05, **P < 0.01). Significant reductions of both types of inclusions were observed only in the right striatum and primary motor cortex. The ratio of pSyn (pSer129)-positive cell density to the average of that measured in the PBS-treated group is shown. **e** Quantification of pSyn (pSer129 #64) protein in the sarkosyl-insoluble fraction from the right anterior cortex 30 days after intrastriatal inoculation of PFFs (n = 4 per each group). The pSyn levels are shown as the ratio of the band signal to the average of that measured in the PBS-treated group. “Anterior cortex” means the anterior half of the cortex. The “motor (sensory) cortex” corresponds to the primary motor (somatosensory) cortex. ND means not detected. All the data are expressed mean ± SEM

To evaluate the effect of local *Snca* ASO pre-treatment far from the aSyn PFFs inoculation site, we then injected *Snca* ASOs into the right striatum, which was previously shown to not downregulate aSyn protein in the contralateral striatum (Fig. 1f), 2 weeks prior to PFFs inoculation into the left striatum (Fig. 4a). pSyn immunostaining showed significant reductions in the density of pSyn-positive somatic and neuritic inclusions only in the right striatum (by approximately 88% for somatic inclusions and 89% for neuritic inclusions) and right primary motor cortex (by approximately 63% for somatic inclusions and 54% for neuritic inclusions) in the vicinity of the *Snca* ASO injection site, compared to the non-treated (PBS) group (Fig. 4b–d). A significant reduction was also observed in the left substantia nigra (by approximately 75% for somatic inclusions and 68% for neuritic inclusions) (Fig. 4c, d). Immunoblotting showed a tendency toward pSyn levels reduction in the sarkosyl-insoluble fraction from the anterior half of the cortex on the ipsilateral side of *Snca* ASO injection (Fig. 4e).

## Discussion

Our study demonstrates that suppression of endogenous *Snca* gene expression by pre-treatment with *Snca* ASOs at the aSyn PFFs inoculation (seeding) site prevented the progression of various pathological changes, including the appearance of pSyn pathology in the whole brain and abnormal pSyn insolubilization in our mouse aSyn pathological transmission models of PD and DLB. In addition, post-treatment with *Snca* ASOs at the seeding site reversed the progression of pSyn pathologies and the abnormal pSyn insolubilization. Finally, pre-treatment with *Snca* ASOs at the site contralateral to the seeding site suppressed the pSyn pathological propagation limitedly in areas within the vicinity of the ASO injection site without inhibiting the propagation to the other areas. These results demonstrate the potential of *Snca* ASOs as a therapy for PD and DLB patients.

As in previous reports, the pSyn pathologies spread throughout the brain over time after the inoculation of synthetic PFFs (seeds) generated from mouse recombinant aSyn into the striatum of WT mice [28, 49, 54, 58]. These pathologies had spread to areas with both direct neural connections and networks through multiple connections to the left striatum. It is also known that callosotomy can prevent the appearance of pSyn pathologies in the contralateral striatum and cortex of a similar model [58]. These data suggest that the transmission of pSyn aggregate amounts depended on the connectivity. Immunostaining of anti-ubiquitin and anti-p62 antibodies were also positive in these pathologies. Interestingly, these pSyn pathologies were stained by Campbell-Switzer silver staining, not Gallyas-Braak staining, similar to what is observed in human PD [11] and DLB [67] patients. Campbell-Switzer-positive staining has been previously reported in the brains of aged homozygous transgenic mice with human A53T mutation (M83 mice) and M83 mice inoculated with brain extracts from PD or MSA patients [44]. However, there is no report of this pathology in WT mice inoculated with recombinant mouse aSyn PFFs, indicating that this model has a high pathological similarity to PD patients.

**Fig. 5:**
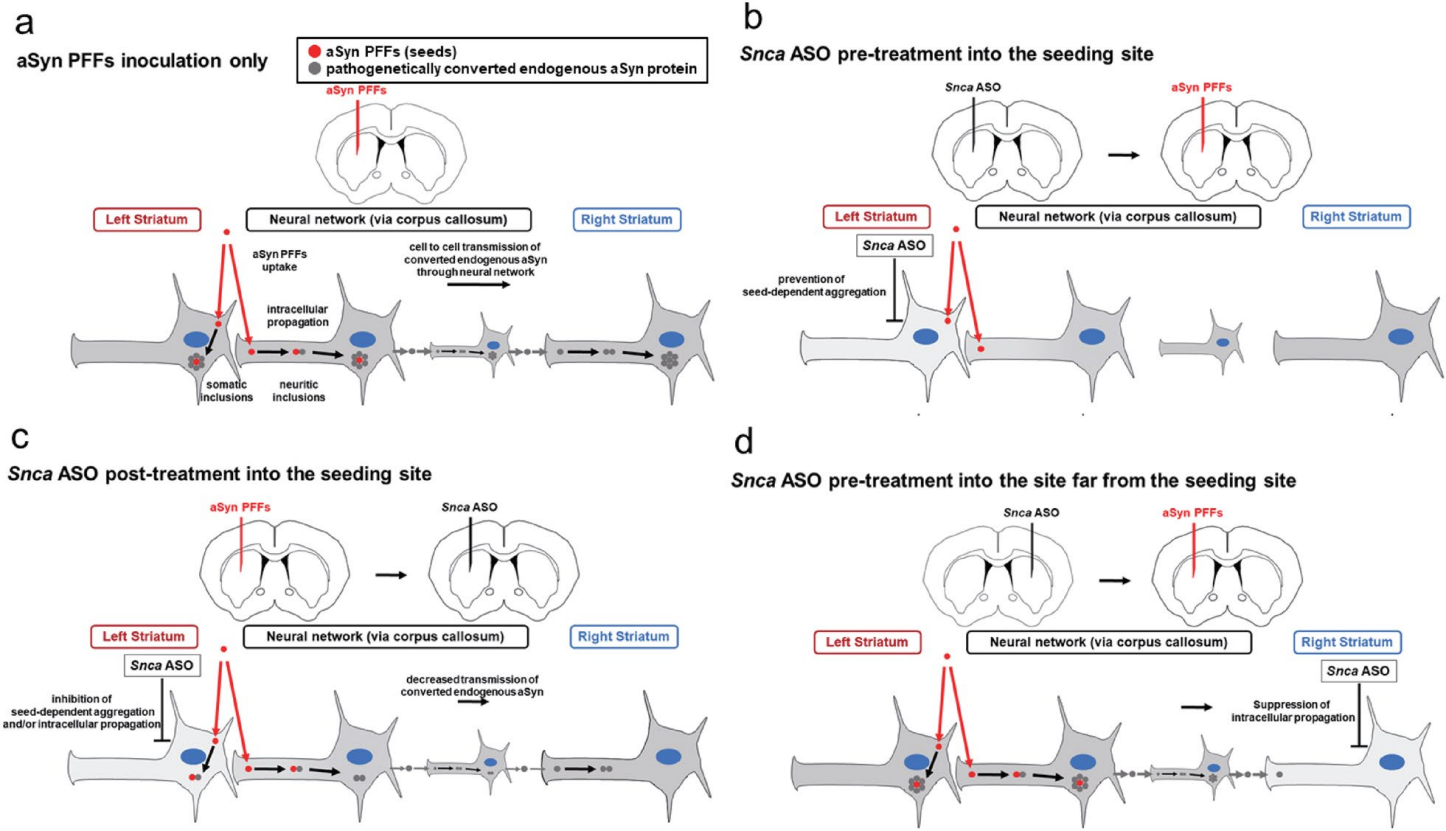
Different effects on aSyn pathology propagation depending on timing and location of local *Snca* ASO injection. **a** Hypothetical model for the propagation of aSyn pathologies caused by the topical injection of aSyn PFFs as seeds. The inoculated seeds are uptaken (seeding) into neurons at the site of inoculation or into neurons whose axons project to them. Seeds convert endogenous aSyn into the pathogenetic structure and form inclusions (seed-dependent aggregation). The inclusions propagate from the neurite to the soma by recruiting more endogenous aSyn (intracellular propagation). Pathological propagation from cell to cell is caused by pathogenetically-converted endogenous aSyn transmission through neural networks and subsequent intracellular propagation (network propagation). **b** *Snca* ASO pre-treatment into the seeding site may prevent the initiation of seed-dependent aggregation by reducing the amount of endogenous aSyn prior to seeding. **c** *Snca* ASO post-treatment into the seeding site may inhibit the seed-dependent aggregation and/or intracellular propagation from the neurite to the soma in the neurons of the seeding site and those projecting to them by reducing the amount of endogenous aSyn and pathogenetically-converted aSyn production after seeding and decrease subsequent network propagation to the sites far from seeding. **d** *Snca* ASO pre-treatment into the site far from the seeding may suppress network propagation to the site by locally reducing the amount of endogenous aSyn and intracellular propagation in neurons. Intact endogenous aSyn protein is shown in gray color in the neuronal background and its reduction is represented by a decrease in color intensity

*Snca* ASO pre-treatment into the same site of seeding prevented the aggregation of pSyn in the substantia nigra on the inoculation side, similarly to previous reports using the same model with ICV injection of ASOs against *Snca* mRNA [20]. In addition, the spread of pathological changes at most ipsilateral and contralateral sites was almost suppressed. One explanation for the complete prevention of pSyn pathologies and the abnormal aSyn insolubilization of the ipsilateral lesion with direct connectivity to the striatum may be that the suppression of endogenous aSyn by ASOs inhibited the initiation and subsequent intracellular propagation of pSyn pathologies induced by the uptaken PFFs. In the contralateral (right) brain area with connectivity to the left striatum via multiple neural connections, although there was no aSyn protein level reduction by ASOs, the absence of intracellular propagation of pSyn in the left striatum and transmission of PFFs via multiple neural connections may result in the lack of pathological changes (Fig. 5a, b). On the other hand, slight pSyn pathology was observed only in the contralateral (right) primary somatosensory cortex and amygdala, which have direct neural connectivity to the left striatum. It means that only PFFs were uptaken directly by the presynaptic terminals and transported retrogradely, with the lack of ASO effects resulting in progressive pSyn aggregation. However, the pathology might be less severe because of the lower neuronal connectivity to the contralateral than to the ipsilateral regions [2]. In this model, it is suggested that PFFs convert the endogenous aSyn protein in the first neuron where it is taken up, and it is the converted endogenous aSyn, not PFFs, that transmit throughout the brain and then propagate intracellularly.

Although previous studies have shown that *Snca* knockout neurons [85] and mice [49] do not exhibit abnormal aSyn PFFs-induced pathological aggregates, it is noteworthy that most of the pathologies were suppressed despite a 60% reduction in aSyn proteins at the time of PFFs injection. This suggests that the pathologies are treatable by suppressing endogenous aSyn proteins by 60% to inhibit intracellular propagation. Thus, there might be a required threshold of endogenous aSyn protein levels for the initiation and intracellular propagation of the pSyn pathologies induced by exogenous aSyn seeds. These results are consistent with a previous report that inoculation of mouse-derived PFFs in *Snca* heterozygous knockout mice with a reduction of about 30% in aSyn proteins [1] resulted in the very slow progression of the disease [49]. Since there have been previous studies showing abnormal synaptic function in the substantia nigra [1] and psychiatric symptoms [60, 61] in *Snca* knockout mice, quantitively and spatially limited endogenous aSyn reduction by ASO may be suitable as a treatment for synucleinopathies.

Simultaneous injection of ASOs also showed a significant reduction of the pSyn-positive somatic and neuritic inclusions 30 days after PFFs inoculation in many brain areas. Although it is expected to take a few days for the reduction of aSyn protein levels by ASOs, simultaneous ASO injection still completely suppressed both pathological changes except in the primary somatosensory cortex and amygdala on the side contralateral to the seeding site same as in the pre-treatment experiment. pSyn aggregation is initiated in neurons after the uptake of PFFs; however, the pathological changes might be mitigated by the interrupted supply of endogenous pathologically converted aSyn protein. In addition, when ASOs were injected 7 and 14 days after PFFs inoculation, there was no decrease in the neuritic inclusions; however, a significant reduction was observed in the somatic inclusions. It is known that in the brain of PD patients, LNs precede LBs [15, 22, 86], with another study suggesting structural continuity between them [36]. Similarly, in the mouse model, pSyn aggregation in the primary neuron progresses gradually from the neurite to the soma over time as PFFs get absorbed from the nerve terminal [52]. This neuritic-to-somatic inclusion process crosses the neurodegeneration threshold and is considered to reach pathological significance. In addition, the pSyn protein levels in the sarkosyl-insoluble fraction were drastically suppressed. Despite the increase in the number of neuritic inclusions, there was little increase in the pSyn protein levels in the sarkosyl-insoluble fraction. A report that the Lewy body is likely to be located near the voluminous Lewy neurite suggests a quantitative correlation between Lewy pathologies and somatic inclusions [12]. Surprisingly, ASOs injected 7 and 14 days after seeding showed improvement in the pSyn pathologies as shown by immunoblotting, with no difference between the two injection times. Our data suggest that once established, the aSyn pathologies improved, as previously reported [20]. Taken together, the fact that the pathology was localized only in neurites suggests that 7- and 14-days post-treatment with ASOs was sufficiently effective and that pathogenic aSyn aggregate deposition is reversible. For the first time in vivo, we show that suppression of endogenous aSyn expression levels by *Snca* ASOs prevents morphological progression from earlier neuritic inclusions to larger, more mature somatic inclusions (Fig. 5c).

The question was raised as to whether suppression of endogenous aSyn levels by ASOs might not prevent the formation of somatic and neuritic inclusions if a large amount of pathological aSyn was continuously transmitted. In addition, when ASOs are injected by ICV as reported in a previous study [20], it is difficult to distinguish whether the intracellular propagation from the inoculated PFFs at the inoculation site or the transmission of pathological misfolded endogenous aSyn is prevented since endogenous aSyn levels are suppressed throughout the brain. Therefore*,* we injected a *Snca* ASO pre-treatment (14 days) into the contralateral (right) striatum, where multiple neural connections are from the left striatum (seeding site). At 30 days after PFFs inoculation (44 days after ASO injection), the pSyn pathology had spread to many areas of the brain. In the right striatum, the site of ASO injection, the formation of somatic and neuritic inclusions was almost completely suppressed, as was their formation in the nearby primary motor cortex, where there also was a reduction in pSyn protein levels in the sarkosyl-insoluble fraction. The suppression of pathology in these areas correlated with the degree of endogenous aSyn protein suppression. These results suggest that the presence of endogenous aSyn is more important for intracellular propagation than the amount of abnormally-converted aSyn transmitted from other neurons and that ASOs might prevent the propagation from transmitted endogenous misfolded aSyn (Fig. 5d).

One limitation of this study is the short duration of observation, limited to 30 days. In the similar mouse model of recombinant mouse aSyn PFFs intrastriatal inoculation, neuronal death, including TH positive neurons in the substantia nigra [49], was observed 90–180 days after inoculation, and the deterioration in motor function [49, 54]was reported at the same time. Consequently, our study could not assess the potential preventive or therapeutic impact of Snca ASO over such an extended timeframe.

## Conclusion

We demonstrate that local injection of *Snca* ASOs achieved a strong reduction in endogenous aSyn around the site of injection. In the aSyn pathological propagation model mice, ASO injection into the seeding site prevented or ameliorated the initiation and propagation of pSyn pathologies to both the site and other brain areas with neural connections to the site in a time-dependent manner and reduced other associated pathologies. Biochemically, the insolubilization of aSyn was also inhibited. *Snca* ASOs injection via multiple neural connections from the seeding site also inhibited the propagation of pSyn pathologies to the ASO injection site. These results indicate that ASO-induced reduction in endogenous aSyn has preventive, disease-modifying, and curative potential in all types of synucleinopathies.

### Supplementary Information

**Additional file 1**. Supplementary Information.
