# Supplementary material for: Effects of local reduction of endogenous α-synuclein using antisense oligonucleotides on the fibril-induced propagation of pathology through the neural network in wild-type mice
Source: Acta Neuropathol Commun. 2024 May 14;12:75. doi: 10.1186/s40478-024-01766-3 (PMC11092238; doi:10.1186/s40478-024-01766-3)
Supplement: Supplementary file 1 — Additional file 1. Supplementary Information. [file 40478_2024_1766_MOESM1_ESM.pdf]

Supplementary Information

Effects of local reduction of endogenous α-synuclein using antisense oligonucleotides on the fibril-induced propagation of pathology through the neural network in wild-type mice

Tatsuhiko Sano[1,2], Tetsuya Nagata[1,2], Satoe Ebihara[1,2], Kie Yoshida-Tanaka[1,2], Ayako Nakamura[1,2], Asuka Sasaki[1,2], Aki Shimozawa[4], Hideki Mochizuki[3], Toshiki Uchihara[1,2], Masato Hasegawa[4], Takanori Yokota[1,2]

Affiliations

[1]Department of Neurology and Neurological Science, Graduate School of Medical and Dental Sciences, Tokyo Medical and Dental University, 1-5-45 Yushima, Bunkyo, Tokyo 113-8519, Tokyo, Japan

[2]Center for Brain Integration Research, 1-5-45 Yushima, Bunkyo, Tokyo 113-8519, Tokyo, Japan, Tokyo Medical and Dental University, Tokyo, Japan

[3]Department of Neurology, Osaka University Graduate School of Medicine, 2-2 Yamadaoka, Suita 565-0871, Japan

[4]Department of Neuropathology and Cell Biology, Tokyo Metropolitan Institute of Medical Science, 2-1-6 Kamikitazawa, Setagaya-ku, Tokyo 156-0057, Japan

Journal name

Acta Neuropathologica Communications

*Correspondence to: Dr. Takanori Yokota and Dr. Tetsuya Nagata

Department of Neurology and Neurological Science, Graduate School of Medical and Dental Sciences,

Tokyo Medical and Dental University, 1-5-45 Yushima, Bunkyo, Tokyo 113-8519, Japan.

TEL: +81-3-5803-5234, FAX: +81-3-5803-0169

E-mail addresses: tak-yokota.nuro@tmd.ac.jp and t-naga.nuro@tmd.ac.jp

Supplementary Fig.1

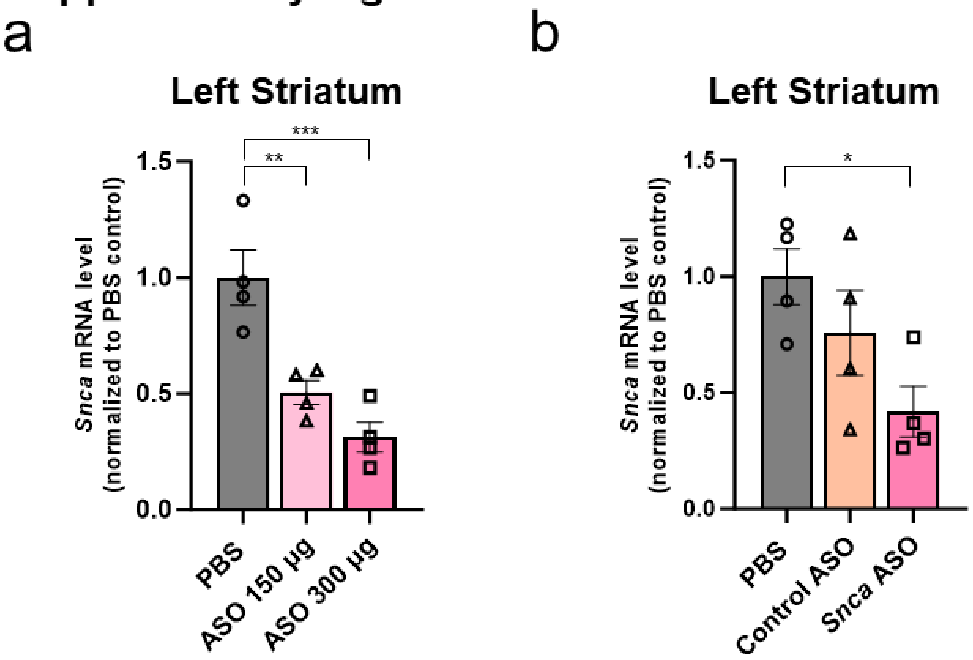

Supplementary Fig.1 Intrastriatal injection of *Snca* ASOs efficiently downregulated the levels of endogenous *Snca* mRNA. a *Snca* mRNA expression levels from the ipsilateral striatum at 7 days after intrastriatal injection of PBS, 150 µg *Snca* ASO or 300 µg *Snca* ASO by qRT-PCR (n=4 per each group; $^{**}P < 0.01$, $^{***}P < 0.001$). **b** *Snca* mRNA expression levels from the ipsilateral striatum at 14 days after intrastriatal injection of PBS, 300 µg Control ASO or 300 µg *Snca* ASO by qRT-PCR (n=4 per each group; $^{*}P < 0.05$). All data are expressed mean ± SEM.

Supplementary Fig.2

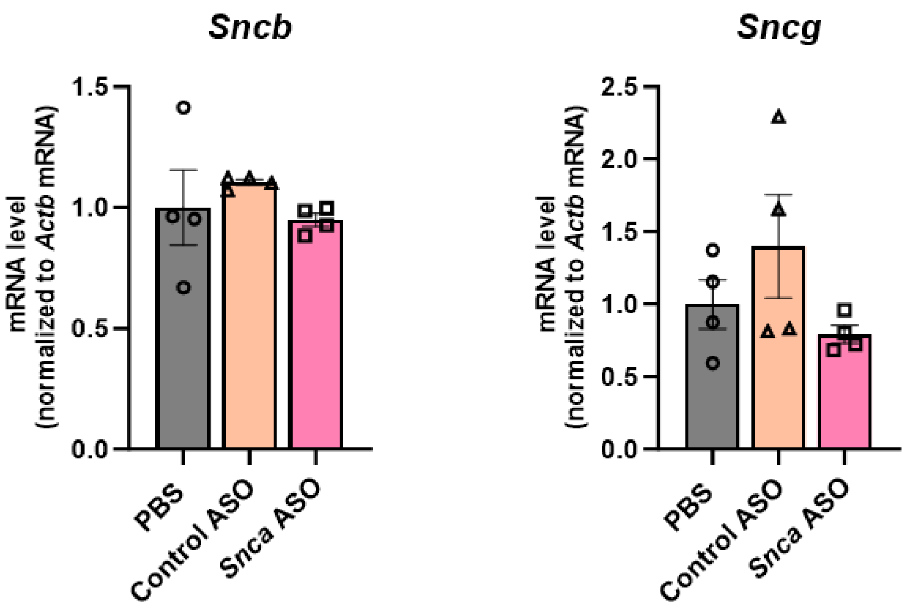

Supplementary Fig.2 Intrastriatal injection of *Snca* ASOs did not affect the levels of *Sncb* and *Sncg* mRNA. *Sncb* and *Sncg* mRNA expression levels from the ipsilateral striatum at 14 days after intrastriatal injection of PBS, 300 μg Control ASO, or 300 μg *Snca* ASO by qRT-PCR (n=4 per each group). All data are expressed mean ± SEM.

Supplementary Fig.3

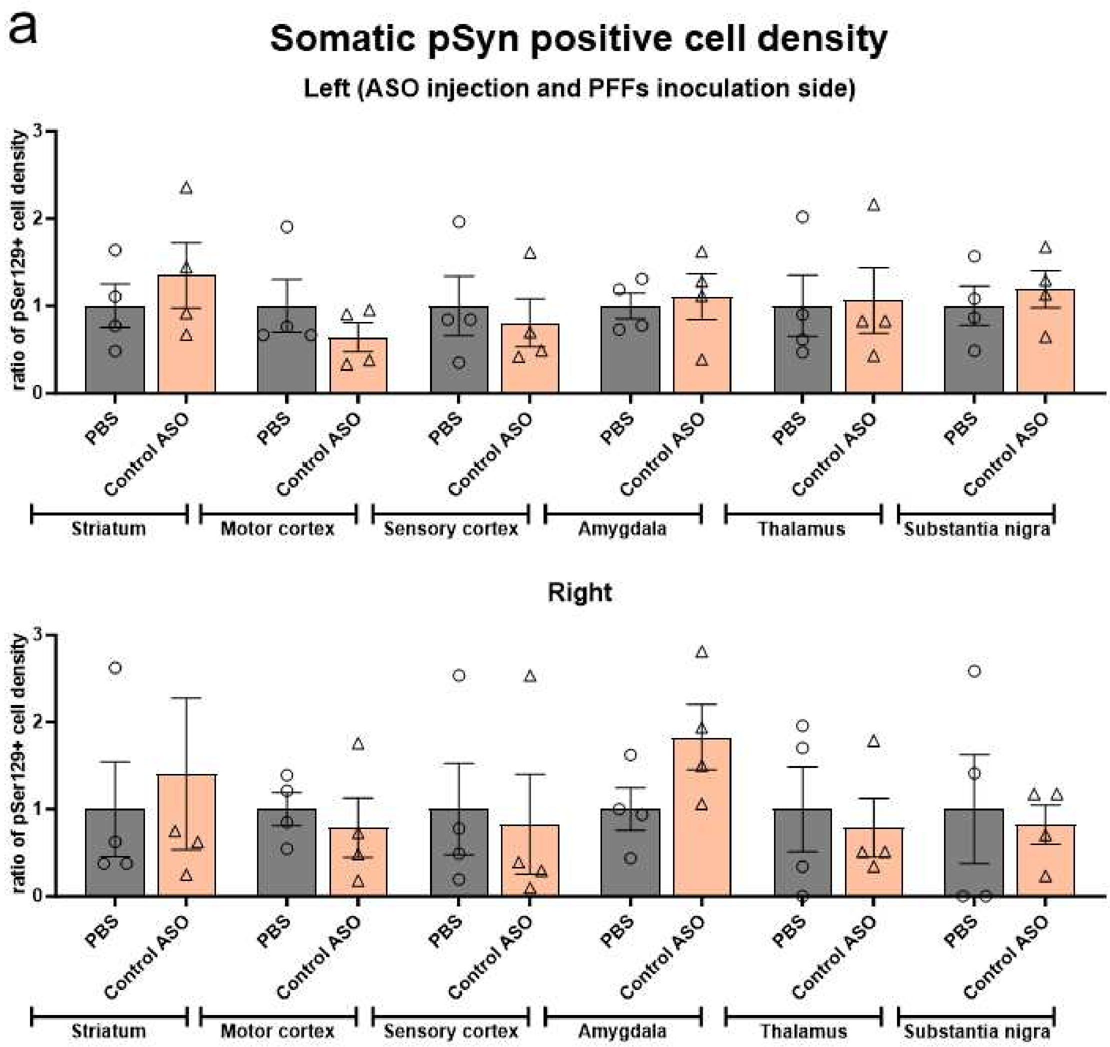

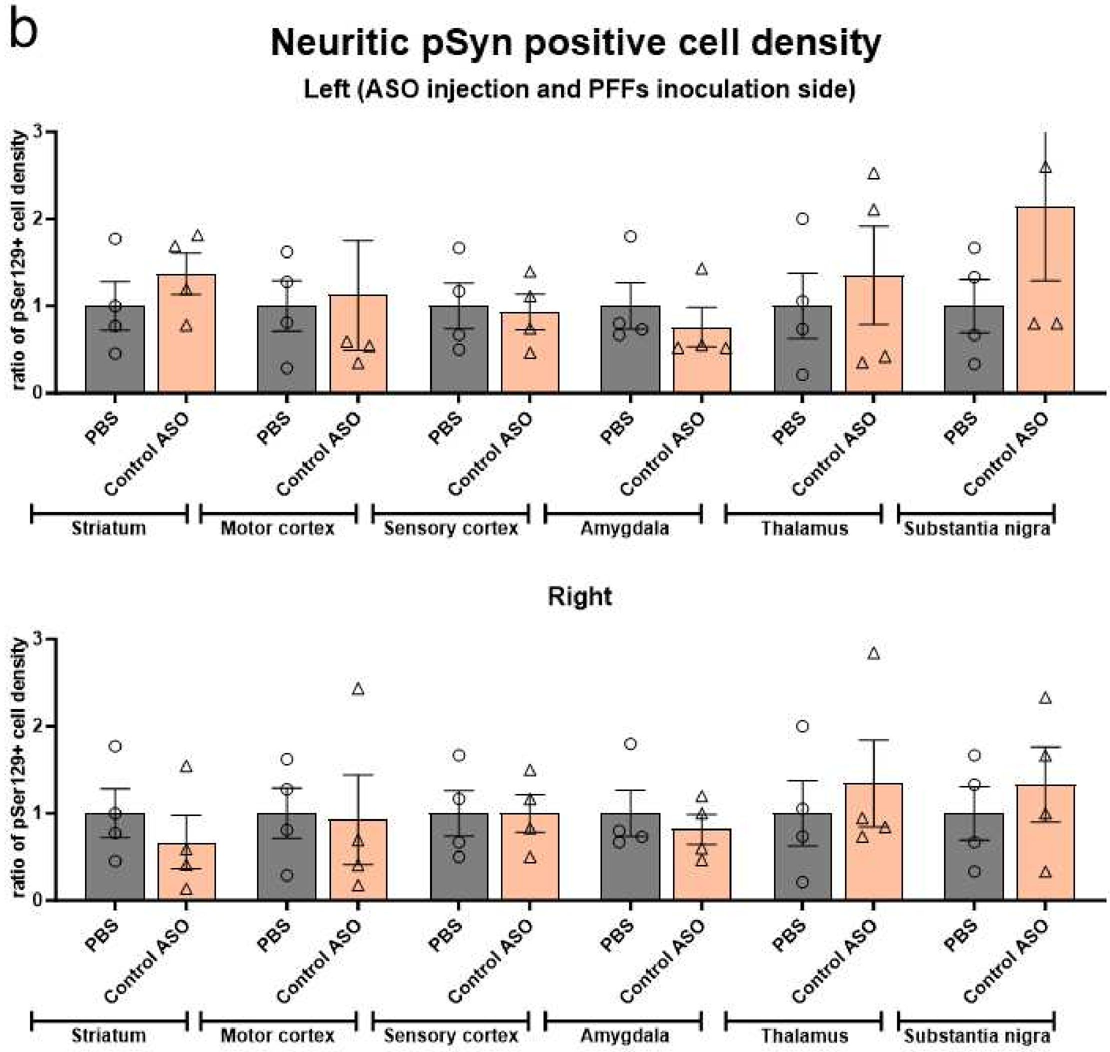

Supplementary Fig.3 Comparison of pSyn pathologies between the PBS ipsilateral pre-treatment and the Control ASO ipsilateral pre-treatment groups in WT mice with aSyn PFFs intrastriatal inoculation. The ratio of cells with pSyn (pSer129) positive somatic (**a**) and neuritic (**b**) inclusions density in the striatum, motor cortex, sensory cortex, amygdala, thalamus, and substantia nigra on each side of the brain (n=4 per each group). The ratio of pSyn (pSer129) positive cell density to the average of that from the PBS-pre-treated group is shown. The "motor (sensory) cortex" corresponds to the primary motor (somatosensory) cortex. All data are expressed mean ± SEM.

Supplementary Fig.4

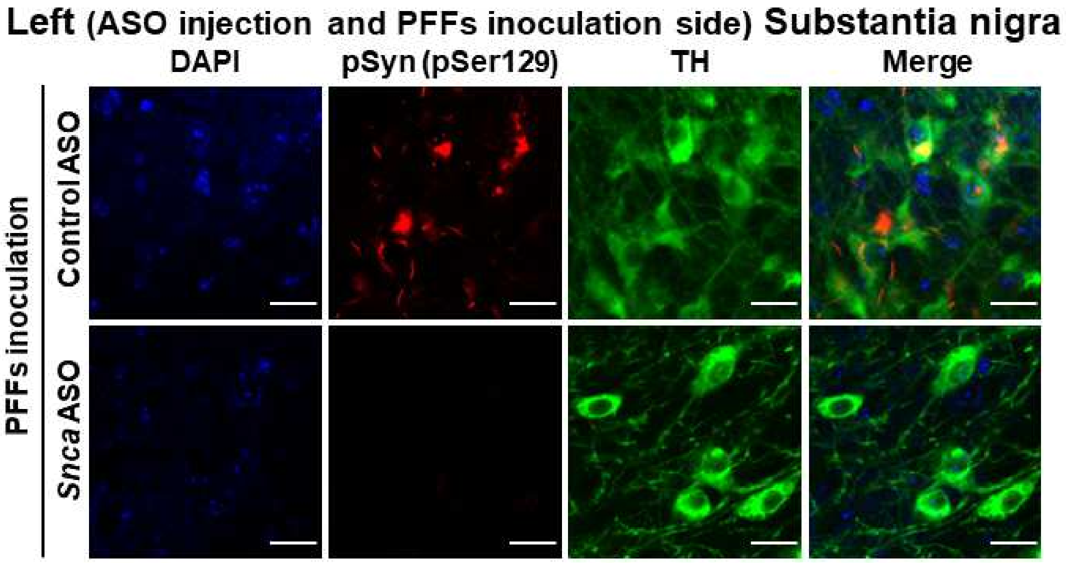

Supplementary Fig.4 Intrastriatal ipsilateral pre-treatment of *Snca* ASOs prevents the appearance of pSyn pathologies in the tyrosine hydroxylase (TH) positive neurons of ipsilateral substantia nigra. Representative images of double-labeled immunofluorescence with pSyn (pSer129) and TH in the left substantia nigra. DNA is stained with DAPI. The scale bar represents 20 µm.

Supplementary Fig.5

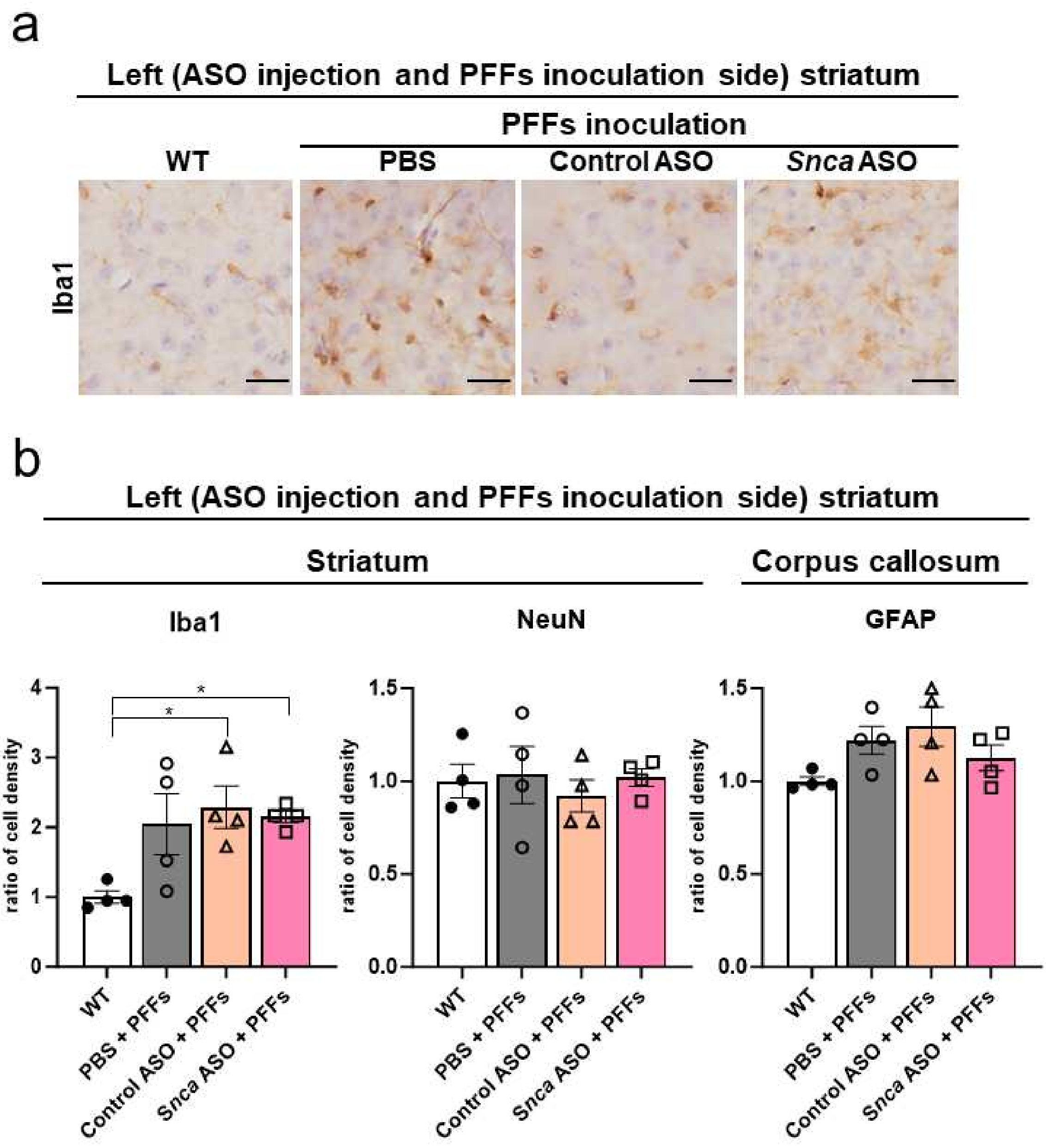

Supplementary Fig.5 ASO ipsilateral pre-treatment did not affect the cell viability and host immune response. a Representative images of Iba1-positive cells in the left striatum wild-type (WT) mice 30 days after PFFs inoculation and PBS, Control ASO or *Snca* ASO injection 14 days before. The scale bar represents 20 µm. **b** The ratio of Iba1- and NeuN- positive cell density in the left striatum and

the ratio of GFAP- positive cell density in the left corpus callosum. The ratio of cell density to the average of that measured in the WT group is shown ($n = 4$ per each group; *$P < 0.05$). The density of Iba1-positive cells equally increased in the PFFs inoculation groups compared to the WT group. All data are expressed mean ± SEM.

Supplementary Fig.6

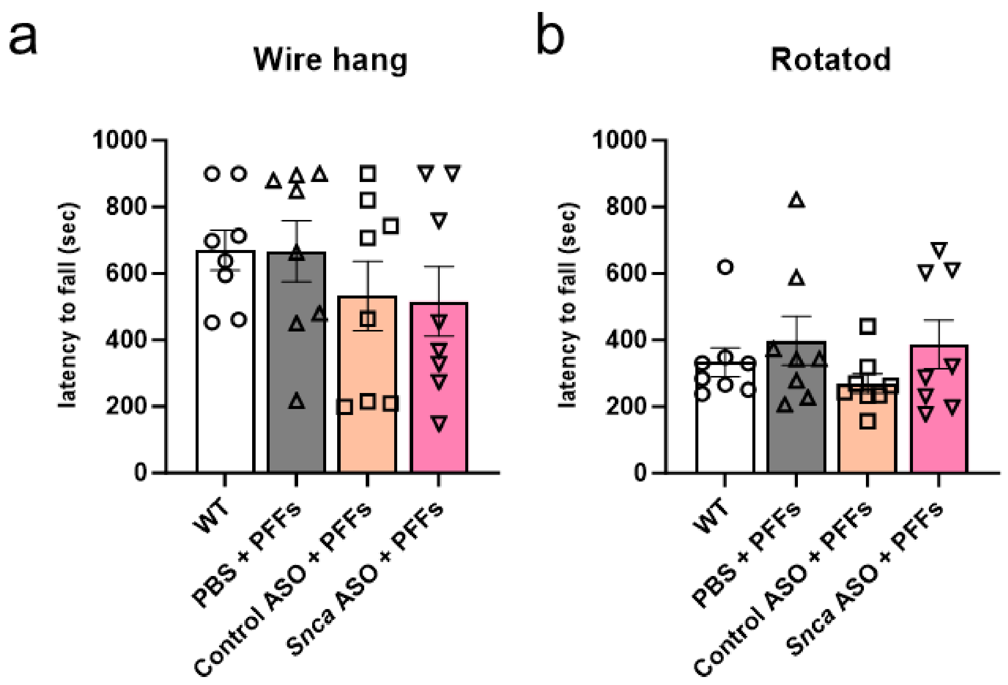

Supplementary Fig.6 ASO ipsilateral pre-treatment did not affect the motor function. a Wire hang test (**a**) and rotarod tests (**b**) of wild-type (WT) mice and 30 days after PFFs inoculation and PBS, Control ASO or *Snca* ASO injection 14 days before (n=8 per each group). All data are expressed mean ± SEM.

Supplementary Table.1 ASOs used in this study.

	Sequence (5′–3′)
Snca ASO	CCAACATTTGTCACTTGCTC
Control ASO	ATCCTTCTATCGGTCACTCA

Note: Red font indicates 2′-O-methoxyethyl (MOE); black font indicates DNA; all phosphorothioate (PS) backbone; A, adenine; ASO, antisense oligonucleotide; C, cytosine; C in the wing portion of ASO, 5-methylcytosine; G, guanine; U, uracil.

Supplementary Table.2 Primary antibodies used in this study.

Primary Antibodies	Type	Source	Dilution
Anti-aSyn (Synuclein-1)	Mouse monoclonal	BD Transduction Laboratories (Clone 42)	1:1000
Anti-phosphorylated aSyn (pSer129)	Rabbit monoclonal	Abcam (ab51253)	1:2000
Anti-phosphorylated aSyn (pSer #64)	Mouse monoclonal	FUJIFILM Wako Pure Chemical Corporation (#64)	1:1000
Anti-tyrosine hydroxylase	Mouse monoclonal	Millipore (MAB318)	1:2000
Anti-p62	Guinie pig polyclonal	PROGEN (GP62-C)	1:1000
Anti-ubiquitin	Rabbit monoclonal	Abcam (ab140601)	1:15000
Anti-NeuN	Chicken polyclonal	Millipore (ABN91)	1:3000
Anti-GFAP	Goat polyclonal	Abcam (ab53554)	1:1000
Anti-Iba1	Rabbit polyclonal	FUJIFILM Wako Pure Chemical Corporation (019-19741)	1:1000
Anti-phosphorothioate	Rabbit polyclonal	Ionis Pharmaceuticals	1:15000
Anti-β-actin (HRP conjugated)	Mouse monoclonal	Proteintech (HRP-60008)	1:10000

Supplementary Table.3 Primers and probe used in this study.

Gene	TaqMan probe (Applied Biosystems)
**Mouse *Snca*** (NM_001042451.2)	Mm00447331_m1
**Mouse *Sncb*** (NM_033610.2)	Mm00504325_m1
**Mouse *Sncg*** (NM_011430.3)	Mm00488345_m1

Gene	Forward	Rerverse	Probe
**Mouse *Actb*** (NM_007393.5)	CGCGAGCACAGCTTCTTTG	CATGCCGGAGCCGTTGTC	CACACCCGCCACCAGTTCGCCATG
